# Neuroimmunological Implications of Subclinical Lipopolysaccharide from *Salmonella* Enteritidis

**DOI:** 10.3390/ijms19103274

**Published:** 2018-10-22

**Authors:** Anita Mikołajczyk, Dagmara Złotkowska

**Affiliations:** 1 Department of Public Health, Faculty of Health Sciences, Collegium Medicum, University of Warmia and Mazury in Olsztyn, 10-082 Olsztyn, Poland; 2 Department of Food Immunology and Microbiology, Institute of Animal Reproduction and Food Research, Polish Academy of Sciences in Olsztyn, 10-748 Olsztyn, Poland; d.zlotkowska@pan.olsztyn.pl

**Keywords:** LPS from *S*. Enteritidis, neuropeptides, dopamine, CD4 T-lymphocytes, CD8 T-lymphocytes, haptoglobin, cytokines, cervical lymph nodes, prefrontal cortex, substantia nigra

## Abstract

Mounting evidence has indicated that lipopolysaccharide (LPS) is implicated in neuroimmunological responses, but the body’s response to subclinical doses of bacterial endotoxin remains poorly understood. The influence of a low single dose of LPS from *Salmonella* Enteritidis, which does not result in any clinical symptoms of intoxication (subclinical lipopolysaccharide), on selected cells and signal molecules of the neuroimmune system was tested. Five juvenile crossbred female pigs were intravenously injected with LPS from *S.* Enteritidis (5 μg/kg body weight (b.w.)), while five pigs from the control group received sodium chloride in the same way. Our data demonstrated that subclinical LPS from *S.* Enteritidis increased levels of dopamine in the brain and neuropeptides such as substance P (SP), galanin (GAL), neuropeptide Y (NPY), and active intestinal peptide (VIP) in the cervical lymph nodes with serum hyperhaptoglobinaemia and reduction of plasma CD4 and CD8 T-lymphocytes seven days after lipopolysaccharide administration. CD4 and CD8 T-lymphocytes from the cervical lymph node and serum interleukin-6 and tumour necrosis factor α showed no significant differences between the control and lipopolysaccharide groups. Subclinical lipopolysaccharide from *S.* Enteritidis can affect cells and signal molecules of the neuroimmune system. The presence of subclinical lipopolysaccharide from S. Enteritidis is associated with unknown prolonged consequences and may require eradication and a deeper search into the asymptomatic carrier state of *Salmonella* spp.

## 1. Introduction

Our growing knowledge of the role of viruses and bacteria as a cause of mental disorders, cancer, and neurodegenerative and metabolic diseases may help in preventing certain human chronic diseases that pose serious public health problems [1,2,3,4,5]. Endotoxin LPS (lipopolysaccharide) is one of the most important bacterial components contributing to many chronic diseases and sepsis [6]. Lipopolysaccharide animal models for induction of Parkinson’s disease have been used by many researchers [7,8,9,10]. The mechanisms underlying Parkinson’s disease (PD) models indicate that LPS induces microglial activation. LPS inflammation by the activation of glial cells and a series of inflammatory mediators, including proinflammatory cytokines, chemokines, reactive oxygen species, and nitric oxide, plays an important role in neurodegenerative diseases, including PD, Alzheimer’s disease (AD), amyotrophic lateral sclerosis, psychiatric disorders, cognitive impairment, and depression [11,12,13,14]. Recent evidence suggests that LPS may play an important role in some neurodegeneration and metabolic diseases, not only in rodents but also in humans [15,16]. Pretorius et al. [17] point to the potentially important role of even very low LPS concentrations (0.2 ng/L) in healthy individuals in the etiology of PD, and they hypothesized that lipopolysaccharide-binding protein may have a protective role in the context of PD. LPS is probably associated with AD neuropathology in humans because it is able to transit physiological barriers to access the brain [18].

How LPS given peripherally induces its effects on the brain is still not clear. Banks et al. [19] suggested that high doses of LPS, in contrast to low doses, can disrupt the blood brain barrier (BBB). They suggested that at low peripheral doses of LPS, the amount entering the brain is below that needed to directly affect the brain. Only about 0.025% of an intravenously administered dose of LPS from *Salmonella* enterica reaches the brain parenchyma [20]. Despite the low passage of LPS across the BBB, LPS could potentially affect brain function by a release of substances from the periphery that can either cross the BBB; interact with immune cells; or alter BBB permeability and functions of the BBB, for example, through dysfunction of vascular endothelial cells or induction of the synthesis of mediators by the cells of the blood–cerebrospinal fluid barrier (BCSFB) and the BBB. Both the BCSFB and the BBB seem equipped to convey signals to the brain parenchyma in response to a low dose of LPS [21,22]. On the other hand, it is known that LPS or its constituents can persist in various tissues and organs for years [23,24]. A large part of LPS is rapidly cleared from the circulation, but the remaining ~20% of LPS could be bound to immune cells such as monocytes, tissue macrophages, neutrophils, or platelets, and thus could potentially be involved in signalling [25].

Knowledge of asymptomatic *Salmonella* infection and latent carriers is limited, but it is well known that LPS causing symptoms of disease can induce an acute phase reaction in humans and animals and release pro-inflammatory cytokines such as interleukin-6 (IL-6) and tumour necrosis factor α (TNF-α) [26,27,28]. Additionally, haptoglobin (Hp), a positive acute-phase protein in response to IL-6, can modulate the inflammatory response induced by LPS. Hp interacts with both resting and activated CD4 and CD8 T-lymphocytes and can play a modulating role in type 1 and 2 T-helper cells, balancing immune responses. [29,30].

LPS can also influence neuropeptides, which are neuroimmune modulators in the communication between the nervous system and the immune system. A single low dose of LPS of *S.* Enteritidis can modulate main enteric neuropeptides [31]. Neuropeptides are widely distributed and involved in many physiological and pathological processes. Neuropeptides of neural and non-neural origin are released in the lymphoid organs such as lymph nodes (LN) and contribute to the modulation of the function of many immunological cells, including lymphocytes and monocytes. Galanin (GAL), neuropeptide Y (NPY), substance P (SP), and vasoactive intestinal peptide (VIP) represent the neuropeptides most involved in neuroimmune modulation. [32,33,34]. Recent studies have illustrated the importance of immune regulation by neuropeptides through direct effects on and CD4 and CD8 lymphocytes [35].

One of the more important catecholamines in health and disease is dopamine (DA). A growing body of research now suggests that not only neuropeptides, but also catecholamines, serve as a link between the nervous and immune systems during physiological and pathological processes [36]. Among its many roles, DA also plays an important role in the immune system. In the immune system, DA acts upon receptors present in immune cells, especially lymphocytes and can be synthesized and released by immune cells themselves [37]. The presence of DA receptors in immune cells suggests that DA plays a physiological role in the regulation of the immune response, and that its deregulation could be involved in many pathological processes such as autoimmune disorders, schizophrenia, and Parkinson’s disease [38,39]. In addition, a decrease of DA in substantia nigra (SN) can lead to PD, and dysregulation of DA in the prefrontal cortex (PFC) is associated with schizophrenia and other psychiatric disorders [40,41].

DA interactions between the nervous and immune systems may be very important in connection with the discovery of meningeal lymphatic vessels [42,43]. Louveau et al. [43] demonstrated that meningeal lymphatic vessels were capable of carrying leukocytes to the deep cervical lymph nodes (dcLN) and, at later time points, to the superficial cervical lymph nodes (scLN). Additionally, cerebrospinal fluid (CSF) drained from the subarachnoid space along the olfactory nerves to nasal lymphatic vessels and subsequently migrated to the scLN [44,45]. Recent studies have also indicated the critical role of the cervical lymph nodes (cLN) on influencing neuroimmunological reactions [46].

In summary, mounting evidence has highlighted that LPS is implicated in neuroimmunological response, but the knowledge of the body’s responses to subclinical doses of bacterial endotoxin remains poorly understood. Taking into consideration all of the facts mentioned above, we are the first to test the influence of a low single dose of LPS from *S.* Enteritidis, which does not result in any clinical symptoms of intoxication (subclinical LPS), and which can hypothetically take place during, for example, the asymptomatic carrier state of *Salmonella* spp. on selected cells and signal molecules of the neuroimmune system:

(1) Peripheral blood levels of CD4 T-lymphocytes, CD8 T-lymphocytes, IL-6, TNF-α, and Hp
(2) cLN levels of CD4 T-lymphocytes, CD8 T-lymphocytes and neuropeptides such as GAL, NPY, SP and VIP
(3) SN and PFC levels of DA

In our study, animal experiments remain essential, and appropriate animal models are irreplaceable because to expose humans to even such low doses of LPS with unknown prolonged consequences is not ethical in scientific studies. In addition, the study of human active substance is hampered by tissue inaccessibility for biopsy. It should be noted that using the pig as a biomedical model plays a critical role in understanding the physiological and pathophysiological processes in the human body. Experiments that use the pig as a biomedical model have very good repeatability of results and recapitulation of human conditions, because pigs are phylogenetically closer to humans than rodents [47,48].

## 2. Results

**Figure 1:**
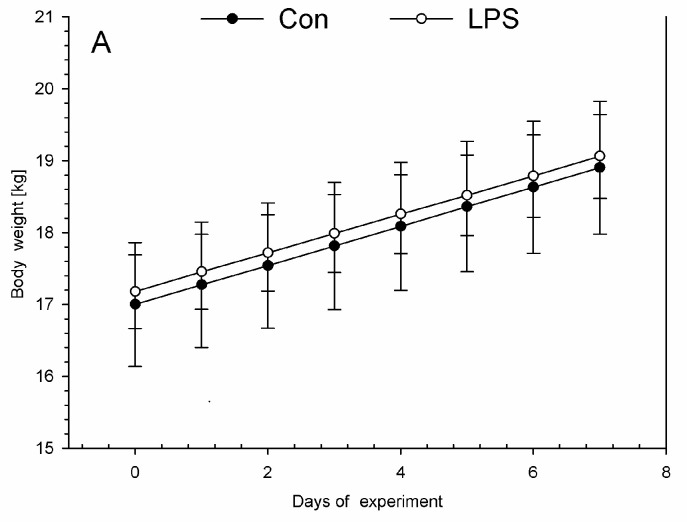

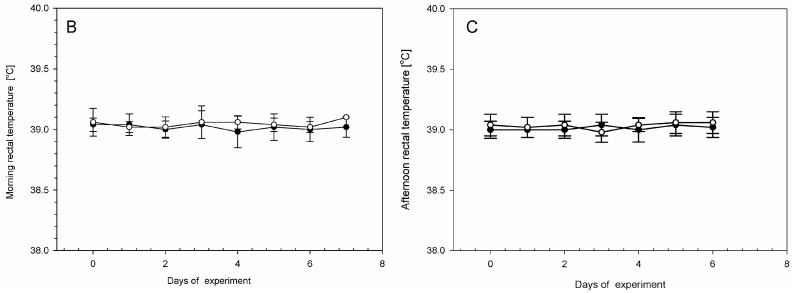
The daily body weights and rectal temperatures in the control group (Con), which received saline, and in the treatment group (LPS), which received lipopolysaccharide (LPS) from *S.* Enteritidis at a dose of 5 μg/kg body weight (b.w.); *n* = 5 pigs/group. (**A**) The body weights were determined once a day in the morning (between 07:00 and 07:30). The first measurement was before premedication and LPS or NaCl administration (day 0), and the final measurement was on the last day of the experiment before antecedent sample collection (day 7). (**B**) The rectal temperatures were measured in the morning (between 07:00 and 07:30). The first measurement was before premedication and LPS or NaCl administration (day 0), and the final measurement was on the last day of the experiment before antecedent sample collection (day 7). (**C**) The rectal temperatures in the afternoon (between 17:00 and 17:30). The first measurement was day 0 (in the day of LPS or NaCl administration), the final measurement was on day 6 of the experiment. The body weight per day and rectal temperatures in the morning and in the afternoon were presented as the mean from group ± SD. The results of rectal temperature and body weight were compared between the control and the LPS group using Student’s *t*-test. The result of rectal temperature and body weight was not statistically significant.

On each day during the experiment, all animals were assessed by a veterinary surgeon as being clinically healthy. All animals in the LPS and control groups were without any symptoms of disease during this investigation. The low dose of LPS *S.* Enteritidis used in this experiment did not evoke any differences in health status, appearance, temperature, or body weight in animals of the LPS group compared with the control group during the entire period of the experiment. Similar body weights with no significant statistically changes were observed between LPS and control group (Figure 1A). Both in the LPS and in the control group, each measurement of the rectal temperature in the morning (Figure 1B) and in the afternoon (Figure 1C) fluctuated in the normal temperature range of pigs. There were no statistically significant differences in the rectal temperature levels between LPS and the control group.

**Figure 2:**
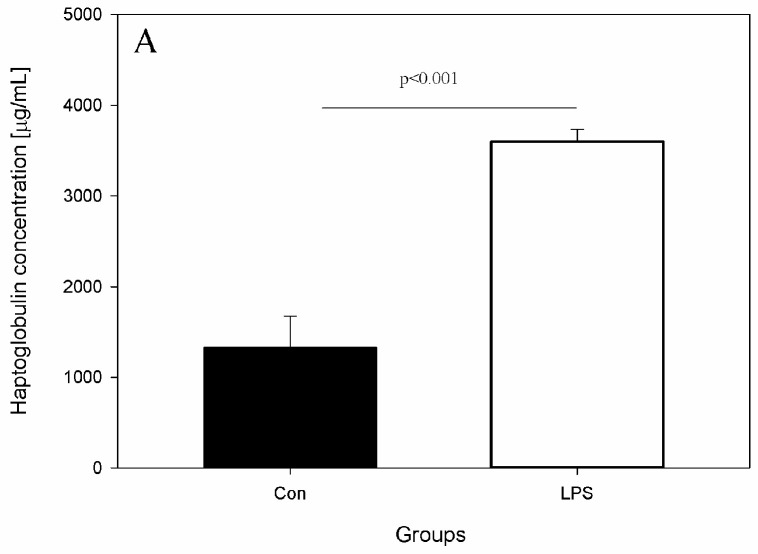

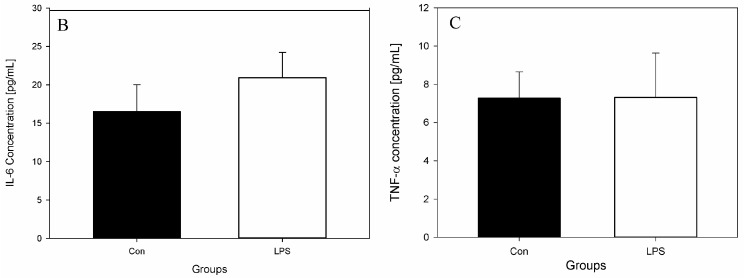
Concentrations of acute phase protein and cytokines in the peripheral blood in the control group (Con), which received saline, and in the treatment group (LPS), which received LPS from *S.* Enteritidis at a dose of 5 μg/kg b.w.; *n* = 5 pigs/group. Bars represent mean ± SD (standard deviation). The statistical analysis was performed by one-way analysis of variance (ANOVA) and Tukey’s tests. (**A**) Serum concentrations of haptoglobin (Hp). The result was statistically different at *p* < 0.001 as compared with the control group (**B**) Serum concentrations of interleukin-6 (IL-6). The result was not statistically significant. (**C**) Serum concentrations of tumour necrosis factor α (TNF-α). The result was not statistically significant.

**Figure 3:**
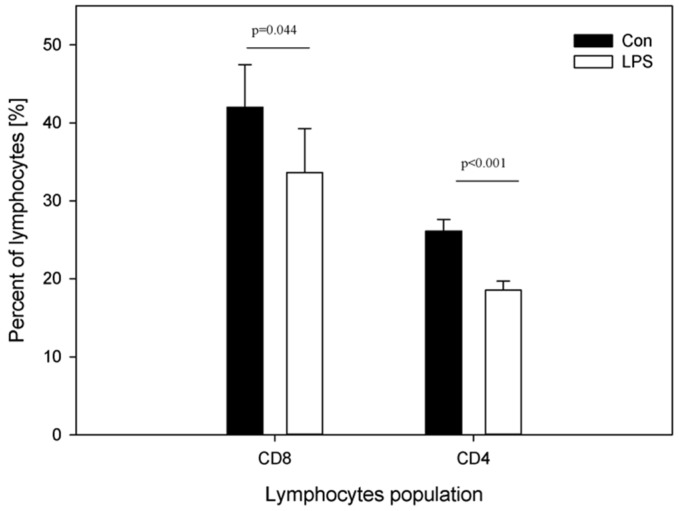

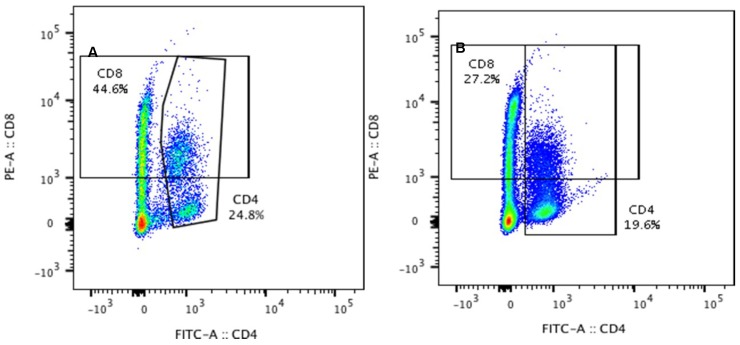
Percentages of CD4 and CD8 T-lymphocytes in the peripheral blood in the control group (Con), which received saline, and in the treatment group (LPS), which received LPS from *S.* Enteritidis at a dose of 5 μg/kg b.w.; *n* = 5 pigs/group. Bars represent mean ± SD (standard deviation). The statistical analysis was performed by one-way ANOVA and Tukey’s tests. Statistically different at *p* = 0.044 (for the percentage of plasma CD8 T-lymphocytes) and *p* < 0.001(for the percentage of plasma CD4 T-lymphocytes) as compared with the control group. Panels A and B present exemplary dot plots with distribution of CD4 and CD8 T-lymphocytes of the control and treatment group. Samples were stained with FITC Anti-Pig CD4a (BD) and PE Anti-Pig CD8a (BD) and analysed on flow cytometer (Beckman Coulter).

**Figure 4:**
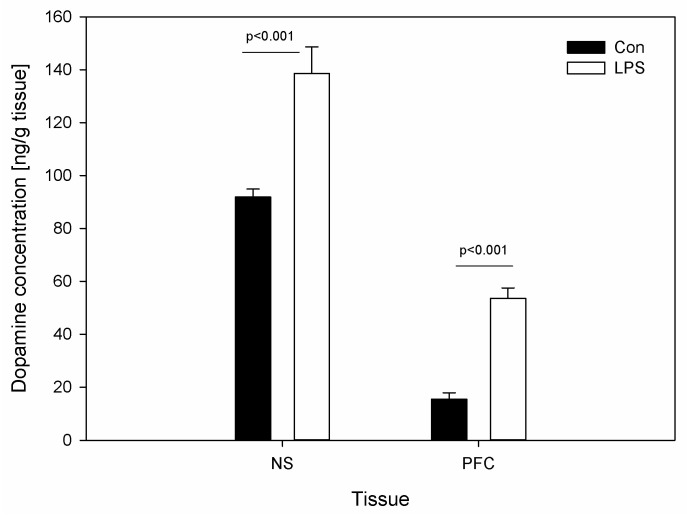
Concentrations of dopamine (DA) in substantia nigra (SN) and in the prefrontal cortex (PFC) in control groups (Con), which received saline, and treatment group (LPS) following the administration of subclinical LPS from *S.* Enteritidis. Bars represent mean ± SD (standard deviation); *n* = 5 pigs/group. The statistical analysis was performed by one-way ANOVA and Tukey’s tests. In both SN and PFC, statistically significant differences of DA concentration for *p* < 0.001 as compared with the control group were observed.

**Figure 5:**
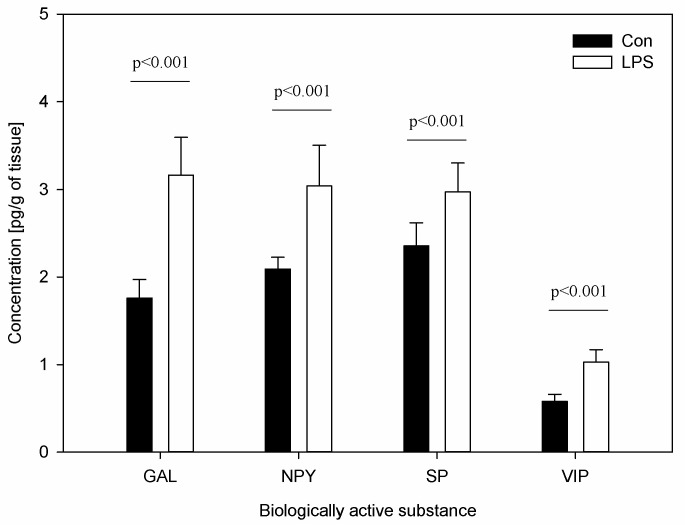
Concentrations of neuropeptides: galanin (GAL), neuropeptide Y (NPY), substance P (SP), and vasoactive intestinal peptide (VIP) in the superficial cervical lymph nodes (scLN). Bars represent mean ± SD (standard deviation); *n* = 5 pigs/group; Con—the control group, which received saline; LPS—the treatment group, which received LPS from *S.* Enteritidis at a dose of 5 μg/kg b.w. (in saline solution). The statistical analysis was performed by one-way ANOVA and Tukey’s tests. The results were statistically different at *p* < 0.001 as compared with the control group.

The effects of LPS on studied levels of parameters and active substances from the blood, scLN, and brain seven days after administration of LPS *S.* Enteritidis are shown in Figure 2, Figure 3, Figure 4 and Figure 5. Figure 2 depicts the levels of Hp, IL-6, and TNF-α in the peripheral blood of animals from the control and LPS group. After the administration of LPS *S.* Enteritidis, pigs had significantly enhanced serum levels of Hp. The level of Hp was almost three-fold higher compared with the control group and amounted to 3599.71 ± 135.69 μg/mL (an increase from 1330.97 ± 344.67 to 3599.71 ± 135.69) (Figure 2A). Although LPS increases serum Hp, no statistically significant changes in serum levels of IL-6 and TNF-α were observed (Figure 2B,C).

Figure 3 depicts the percentage of CD4 and CD8 T-lymphocytes in peripheral blood of animals from the control and LPS group. A decrease in the mean percentage of both plasma CD4 T-lymphocytes from 26.13 ± 1.49 (in control group) to 18.54 ± 1.16 (in LPS group) and plasma CD8 T-lymphocytes from 42.01 ± 5.46 (in control group) to 33.63 ± 5.64 (in LPS group) was noted (Figure 3).

DA levels were determined in the brain tissue. Figure 4 depicts the DA levels in SN and PFC in the control group and the LPS group. Compared with the control, the values of DA increased significantly following the injection of LPS from 91.86 ± 3.05 to 138.58 ± 10.07 ng/g in SN and from 15.41 ± 2.46 to 53.59 ± 3.90 ng/g in PFC (Figure 4).

**Figure 6:**
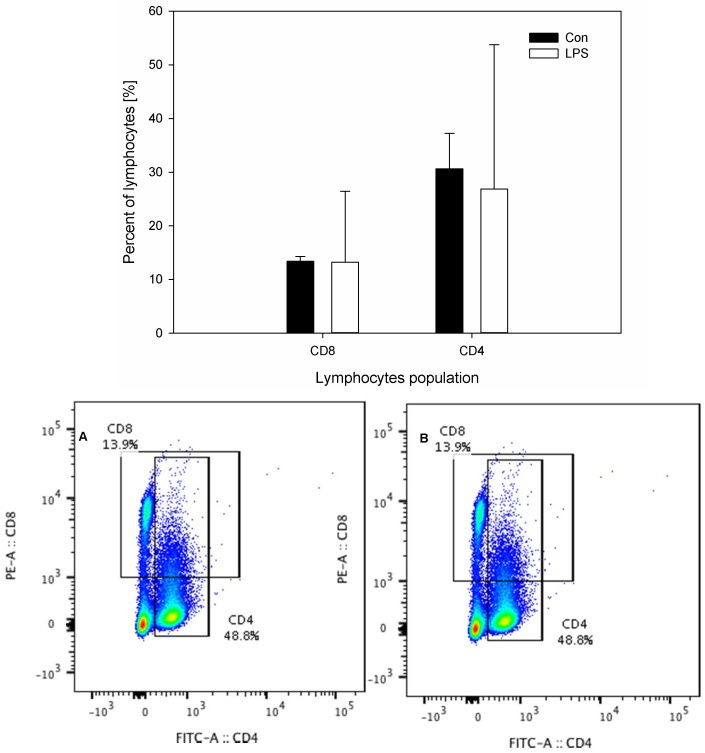
Percentages of CD4 and CD8 T-lymphocytes in the superficial cervical lymph nodes (scLN) in the control group (Con), which received saline, and in the treatment group (LPS), which received LPS from *S.* Enteritidis at a dose of 5 μg/kg b.w.; *n* = 5 pigs/group. Bars represent mean ± SD (standard deviation). The statistical analysis was performed by one-way ANOVA and Tukey’s tests. The result was not statistically significant. Panels A and B present exemplary dot plots with distribution of CD4 and CD8 T-lymphocytes of the control and treatment group. Samples were stained with FITC Anti-Pig CD4a (BD) and PE Anti-Pig CD8a (BD) and analysed on flow cytometer (Beckman Coulter).

The results of the concentration of neuropeptides and the measurements of the percentage of CD4 and CD8 T-lymphocytes in scLN after LPS administration are depicted in Figure 5 and Figure 6. The levels of all studied neuropeptides reached with LPS were significantly greater than in the control group. LPS induced an increased level of GAL (from 1.76 ± 0.21 to 3.17 ± 0.43), NPY (from 2.09 ± 0.14 to 3.04 ± 0.46), SP (from 2.36 ± 0.26 to 2.97 ± 0.33), and VIP (from 0.58 ± 0.08 to 1.03 ± 0.14) (Figure 5). An analysis of the percentages of CD4 T-lymphocytes and CD8 T-lymphocytes in scSN showed no significant differences between the control and the LPS group (Figure 6).

## 3. Discussion

Although the role of LPS as a major pro-inflammatory component released by gram-negative bacteria has been widely established, knowledge of asymptomatic LPS infections remains limited. It may be the case that some of the pathogens or their components, which are able to persist in organisms without causing any symptoms of the disease, can interfere with neural or/and immunological cell functions. To understand the mechanisms of the ability of *Salmonella* spp. to survive in various host cells, the activity of their lipopolysaccharides may be very important for both eradication strategies and the prevention of various disease processes, including neurodegenerative diseases. Additionally, the intra-species and inter-species LPS influence on the immune response control of bacterial infections and LPS biological activity varies [49,50,51]. Even LPS derived from two related species of G-bacteria impacted the regulation of Th-cell responses and T-cell cytokine balances in different ways [52]. Our previous in vitro observations confirmed that LPS shows differences in activity to SP and GAL even within particular serotypes of *Salmonella* spp. [53]. Therefore, considerations of different LPS biological activity depending on various bacterial sources and long-term consequences of LPS different doses must be considered in a research strategy. The ability of LPS to impact cell responses may be attributed to the different bacterial source of LPS, the dosage of LPS, and the time of LPS or the presence of its metabolites in the body. Niehaus [24] reported that 14 years after a laboratory worker developed polyneuropathy, encephalopathy, and parkinsonism after accidental exposure to *Salmonella* Minnesota LPS—the lipopolysaccharides had not been detoxified by the body. Thus, considering the probable neuroimmunological effects of subclinical *S.* Enteritidis LPS with unknown prolonged consequences—it can be seen that asymptomatic *Salmonella* infection and latent carriers are more serious problems than had been assumed [54]. In this study, we investigated the effects of subclinical *S.* Enteritidis LPS in vivo, focusing on the neuroimmune response of Hp, IL-6, TNF-α, and CD4 and CD8 T-lymphocytes in peripheral blood, selected neuropeptides and CD4 and CD8 T-lymphocytes in cLN, and DA in PFC and SN.

Our data demonstrated that seven days after the administration of LPS *S.* Enteritidis, pigs had significantly increased serum levels of Hp, but did not have increased serum levels of TNF-α or IL-6 compared with the control group (Figure 1A–C). Previous studies have used the administration of single or repeated doses of LPS, leading to clinical symptoms of intoxication, which induced short-term increases in serum IL-6 and TNF-α. Qin et al. [55] indicated that a single high dose of systemic LPS in mice led to a decline in the short-term increase of TNF-α to return to the base level by 9 h post treatment, and induced a significant chronic loss of DA neurons beginning at seven months post-treatment. Calvano and Coyle [56] suggested (using the human endotoxin model generated using LPS from *E. coli* O:113 in normal human volunteers) that serum TNF-α and IL-6 peaked at 1.5–2 h after endotoxin administration and again became undetectable after 4 and 6 h, respectively. Similarly, previous studies [57,58] have shown that 12 h after the injection of LPS from *E. coli*, pro-inflammatory cytokines such as IL-6 and TNF-α maintained the same levels as in the control pigs. Despite the lack of differences in the proinflammatory cytokines’ levels in our study, the Hp level was significantly increased. It may be the case that serum levels of Hp were altered in response to subclinical LPS challenges because asymptomatic LPS results in an inflammatory process. It seems likely that subclinical inflammatory activity takes place in the case of such a low dose of LPS with an absence of clinical symptoms of a disease. It is worth noting that, because of the relationship of inflammatory processes with psychiatric and neurodegenerative diseases, Hp is the most frequently studied acute phase protein in major depression and has provided the most consistent results. Hyperhaptoglobinaemia in major depression is significantly related to the activation of cell-mediated immunity from activated T-lymphocytes to monocytes [59]. Major depression patients have abnormal levels of IL-6 and TNF-α, and increased Hp [60]. Robertson et al. [61] found alterations in lymphocyte T subsets without differences in the total numbers of T-cells in patients with multiple sclerosis and with major depression. We have observed a decrease in both plasma CD4 and CD8 T-lymphocytes after subclinical *S.* Enteritidis LPS administration (Figure 3). Juffermans et al. [62] observed a decrease in the number of the fraction of CD4 T-lymphocytes in peripheral blood in healthy volunteers four hours after intravenous administration of LPS from *E. coli*. This is in line with our observations of decreased CD4 T-lymphocytes (Figure 3). However, Juffermans et al. [62] assessed the effects of a single high dose of LPS that caused clinical symptoms of intoxication, rather than the subclinical dose of LPS as was observed in our study. Palmer et al. [63] reported decreased CD4/CD8 T-cell ratios in human subjects without any symptoms of intoxication, but with the presence of subclinical LPS in serum; unfortunately, they did not investigate the source of LPS from the specific bacterial groups that were present in the body.

To our knowledge, there are no prior studies that describe the effect of a single subclinical dose of LPS *S.* Enteritidis on the changes of DA in pig SN and PCF. Our studies have shown that levels of DA in the SN and PFC were significantly greater in the group that received asymptomatic LPS from *S.* Enteritidis than in the control group (Figure 4). Previous studies have described DA levels as a consequence of the inflammation response after *S.* Typhimurium [64] or LPS from *E. coli* [64,65] administration, and that is probably the main reason why they are not in accordance with our results. We used a dose that does not result in any clinical symptoms, as well as LPS from different gram-negative bacteria than that used by the above-mentioned researchers. Guzmán et al. [64] demonstrated that DA levels decreased in hemisphere regions and did not change in cortex regions, the cerebellum, or the medulla oblongata five days after oral administration of *S.* Typhimurium in rats. Another study [65] showed that intrastriatal injection of LPS from *E. coli* in a high dose decreased the content of DA in the rat SN 72 h after an injection. Noworyta-Sokołowska et al. [66] suggested that a high single i.p. LPS from *E. coli* administration does not affect the striatal DA level 180 min after LPS administration, but LPS given repeatedly for five days decreased the DA level. The data indicate that repeated doses and a long period of time are necessary for the progression of inflammation symptoms induced by LPS. Considering the differences between the previous studies and our study, we conclude that subclinical LPS affect DA in a different way than LPS in inducing symptoms of intoxication. It is known that higher levels of DA released in the ventromedial prefrontal cortex play an important role in reward and motivation, and drugs like cocaine, amphetamines, and heroin are highly addictive because they increase the release of DA and then act as DA re-uptake inhibitors. Repeated stressors may also influence the onset of, or relapse to, a number of DA-related disorders [67]. The latest findings [68] show DA dysregulation in different regions of the brain in schizophrenia. Enhancing DA levels in SN during DA treatment for Parkinson’s disease can cause psychotic side effects mimicking the symptoms of schizophrenia. On the other hand, reducing dopaminergic transmission in the treatment of schizophrenia and psychosis can cause Parkinson-like symptoms. Furthermore, data from previous studies [40] have provided evidence for brain DA dysregulation in the schizophrenic brain and confirmed a deficit in DA release in PFC in schizophrenia. Moreover, pathophysiology of the DA synthesis and/or release in mania and in bipolar depression is still inconsistent. Both DA agonists and anti-dopaminergics can improve bipolar depressive symptoms [41]. Hence, finding the hypothetical relationships that may increase DA levels in SN and PFC seven days after subclinical LPS administration with psychiatric or/and neurodegenerative disorders is difficult and requires further research. 

Neuropeptides (like DA), which can be called neuroimmune transmitters, also play neuroimmunological roles as modulatory molecules between immune and nervous cells [69]. Both neuropeptides and DA can be released by neuronal and immunological cells. Under specific stimuli, these cells may release modulatory molecules into the extracellular compartment, thus enabling communications between other cells. As an example, T lymphocytes express neuropeptide receptors for SP and VIP. SP and VIP are released from the lymphoid organs from neuronal and immune cells. At the same time, neural cells express receptors for cytokines, which are released from the immune system and affect neural differentiation. The nervous and immune systems may produce and respond to mediators of immune–neuronal interaction, such as neurotransmitters and cytokines [69]. As we can see, the nervous system is necessary for full immune function and vice-versa, but the abnormal activity of immune or nervous cells may result in disease. In the present study, subclinical LPS increase all examined neuropeptide concentrations in cLN. SP and VIP are the best-studied immunomodulatory neuropeptides. Specific high-affinity receptors for both have been identified on lymphocytes and monocytes, which suggests that they are involved in the direct interaction between immune cells. SP is an immunostimulatory neuropeptide and modulates a number of important immunological functions, including direct effects on T-cell activation [22]. VIP is a well-known anti-inflammatory mediator and may have a dual role in the neuromodulatory system. VIP is generally a suppressive neuropeptide for T-cell proliferation, but can also enhance certain lymphocyte functions by interacting with different VIP receptors and can inhibit activation-induced apoptosis in T lymphocytes [70]. NPY modulates differentiation of T-helper cells and plays an important homeostatic role in balancing disturbances of neuroimmune systems. In addition, NPY is also able to modulate the immunomodulatory effects of other neuropeptides and acts as a neuroimmune transmitter and co-transmitter in neuroimmune crosstalk [33,71]. NPY infusion improves survival during septic shock induced by LPS in mice [72]. The beneficial effects of NPY result from its vasoconstrictor abilities and potentiation of catecholaminergic vasopressor effects, as well as improved critical immune functions [73]. Although the influence of GAL on lymphocytes has not been thoroughly studied, it was found that this neuropeptide has a potent anti-proliferative influence on (at least) certain lymphocyte subpopulations [74]. Considering the above information and our findings of the high level of GAL, NPY, SP, and VIP in cLN seven days after subclinical LPS administration, we hypothesize that dysregulation of neuropeptides is connected with their influence on immune cells. This hypothesis may particularly be confirmed by the fact that in the present study, despite increasing the selected neuropeptides, LPS did not increase either CD4 or CD8 T-lymphocyte levels in cLN. It should be noted that changes in immune cell functions can also influence the expression of neuropeptides in lymphoid organs [75]. Therefore, it is possible that neuropeptides act as a negative feedback inhibitor to decrease CD4 and CD8 T-lymphocyte levels, which have been upregulated in response to LPS.

The observable occurrence of functional interconnections between the nervous and immune systems is inextricably interlinked by recent studies revealing the meningeal lymphatic vessels. The cLN appear to be critical for this neuroimmunological connection [41,42,43,76]. Ligation of the collecting vessels draining to the dcLN resulted in the distension of the dural lymphatic vessels and the accumulation of T-lymphocytes, which suggests that the meningeal lymphatic vessels’ pathway may play a role in antigen presentation and in the movement of peripheral immune cells out of the brain to cLN [42]. Surprisingly, CD4 T-lymphocytes are required for normal learning and memory in the brain, because removal of the dcLN disrupted T-cells circulation and induced learning and memory impairments in mice [77]. Additional experiments have shown that whereas CD8 T-lymphocytes deficiency is negligible, the participation of CD4 T-lymphocytes is important for promoting neurodegeneration of DAergic neurons in the SN of mice undergoing PD [78,79]. The presence of infiltrating immune cells in the central nervous system parenchyma has been detected in most studied neurodegenerative diseases [38,80,81]. Recent evidence suggests that exposure to a high dose of LPS in vitro leads to suppression of CD8 T-lymphocyte proliferation in the mice spleen but not in cLN [82]. The suppression of T-cells in the spleen, but not in the cLN, makes it possible for the continuation of T-cell activation in cLN. Similarly, in the current study, subclinical LPS did not make any direct (or indirect, probably thanks to neuropeptides) changes in the CD4 and CD8 T-lymphocyte levels in comparison with the control group (Figure 6). The presence of receptors for DA, neuropeptides, cytokines, and LPS on T cells in the LN [83] and the possibility of interaction in cLN between CD4 and CD8 lymphocytes and neuropeptides, together with the discovery of meningeal lymphatic vessels, may indicate the existence of relationships that until now have not been considered. A biological factor such as LPS and a pathological process from Alzheimer’s to Parkinson’s disease, schizophrenia, and depression may be linked to a dysfunctional neuroimmune system. Moreover, our study is very interesting in reference to a recent study by Kozina et al. [84], which suggested that peripheral immune signalling plays an unexpected and very important role in the neurodegeneration process.

In our in vivo studies, in comparison with in vitro systems, an account was taken of the normal anatomical distribution of CD 4 and CD8 T-lymphocytes and neuropeptides in LN and DA in the brain and of the integration of the effects of those cells and active substances with cytokines and Hp in blood. Numerous studies investigating the neuroimmunological system have focused on in vitro systems in which concentrations of neuroimmune-active substances could be outside the physiological range and in which no account was taken of the normal anatomical distribution of lymphocytes and neural innervation of lymphoid organs and peripheral and central tissues. Our in vivo study integrated the effect of LPS on the levels of neuropeptides, DA, CD4 T-lymphocytes, CD8 T-lymphocytes, acute phase cytokines, and Hp derived from different tissue and cells following asymptomatic LPS stimulation.

## 4. Materials and Methods

### 4.1. Animal Housing, Health Status, and LPS Administration

All experimental procedures were approved by the Local Ethical Committee for Animal Experimentation in Olsztyn, Poland (decision no.: 73/2015, from 29 September 2015). Animals were kept and treated in accordance with all institutional and national guidelines applicable within the Republic of Poland, as per the Federal Law of 15 January 2015 on Animal Welfare for Science and Education (Dz.U.2015.0.266).

The study was performed on ten juvenile crossbred female pigs (Pietrain × Duroc) and was conducted when the pigs were between eight and nine weeks of age with body weights of 16–18 kg. The animals were maintained for two weeks prior to the experiment in order to allow adaptation to the new environment. The pigs were kept under standard laboratory conditions and fed commercial feed for pigs of this age group. Following assessment by a veterinary surgeon (DVM, Ph.D.), only clinically healthy animals with negative results of analyses of *Salmonella* spp. in faecal samples qualified for the experiment. 

After a two-week adaptive period, pigs included in the experiment were randomly divided into two groups (five pigs each): a control and a treatment group (LPS group). In the morning, animals of both groups were subjected to premedication, according to the method previously described [85], with an intramuscular injection of atropine (Atropinum Sulfuricum Polfa Warszawa S.A., Poland, 0.035 mg/kg b.w.), ketamine (Bioketan, Vetoquinol Biowet Sp. z o.o., Poland and Vetoquinol S.A., France, 7.0 mg/kg b.w.), and medetomidine (Cepetor, CP-Pharma Handelsges mbH, Germany, 0.063 mg/kg b.w.). The premedication of animals allowed accurate and safe (for investigators) injections of LPS. Under premedication, the control animals were injected with 10 mL of 0.9% NaCl (sodium chloride 0.9% WET Baxter, 9 g/1000 mL, Baxter Sp. z o.o. Poland) saline solution into the marginal ear vein, while pigs of the treatment group received lipopolysaccharides from *Salmonella enterica* serotype Enteritidis in the same way (i.e., intravenously) (L7770 Sigma, Aldrich, Germany) at a dose of 5 μg/kg b.w. (in 10 mL saline solution). Such a dose has been previously described as a “low single dose”, which does not result in any clinical symptoms of disease [31,56]. All procedures and drugs were managed and administered by a veterinary surgeon (DVM, Ph.D.). 

During the clinical assessment of the pigs’ health status, conducted by a veterinary surgeon, the observations of the animal care staff were always considered. In addition, during the physical examination, the measurements of temperature and body weight, both in the control and LPS group, were taken into accounts. Pigs were individually weighed once a day in the morning (between 07:00 and 07:30). The body weight data were presented as the mean from group ± SD (standard deviation). Rectal temperature was determined using an animal digital thermometer (model SC 12, SCALA Electronic GmbH, Stahnsdorf, Germany). Temperatures were determined twice daily: in the morning (between 07:00 and 07:30) and in the afternoon (between 17:00 and 17:30) during the seven days after LPS administration. The first measurement was taken before premedication and LPS or NaCl administration, and the final measurement was taken on the last day of the experiment before the antecedent sample collection. The temperature data were presented as the mean from group ± SD. 

### 4.2. Sample Collection

After a seven-day period, which has been described as sufficient for the emergence of changes in the nervous system in previous studies [31,86,87], all animals were pre-medicated (in the above-described manner) and subjected to general anaesthesia using propofol (Scanofol, NORBROOK, Northern Ireland, IRL.PN, 4.5 mg/kg b.w. given intravenously into the marginal ear vein). Blood from the LPS-treated animals and the control group was taken from the vena orbital sinus. Serum was harvested after centrifugation and stored at −80 °C for further analyses of IL-6, TNF-α, and Hp. Heparinised blood samples were collected to isolate peripheral blood mononuclear cells (PBMCs). After euthanasia, with pentobarbital (Morbital, Biowet Puławy Sp. z o.o, Poland, 60–70 mg/kg b.w., given intravenously), the scLN were then isolated [88]. Next, the brains were rapidly removed from the skulls. The SN and PFC were isolated as described by Jelsing et al. [89]. The right dorsal scLN, the SN, and the fragment of PFC were frozen immediately after collection in liquid nitrogen and were stored at −80 °C until processing for further analysis. The left dorsal scLN was used for lymphocyte isolation.

### 4.3. Lymphocytes Isolation Phenotyping

#### 4.3.1. Isolation of Peripheral Blood Mononuclear Cells (PBMCs) from Blood

PBMCs were isolated by gradient centrifugation [90]. An equal volume of phosphate buffered saline (PBS) was mixed with blood. The mixture was layered on Histopaque-1077 (10771, Sigma) at a 3:1 ratio (*v*/*v*) and centrifuged at 512× g/18 °C for 35 min (Eppendorf 5804R, Hamburg, Germany). Mononuclear cells were aspired and washed in incomplete medium (RPMI 1640 cat. no. 11875093; Thermo Fisher Scientific supplemented with 10 mM HEPES (4-(2-hydroxyethyl)-1-piperazineethanesulfonic acid) and 10 units/mL penicillin–streptomycin solution, Waltham, MA, USA), centrifuged at 413× *g*/10 °C for 10 min, and suspended in 1 mL of incomplete medium. The number of lymphocytes was calculated using a Burker Cell Counter after Trypan Blue staining. 

#### 4.3.2. Lymphocytes Isolation from the scLN

The scLN were dounced in incomplete medium and filtered through an 80 µm nylon filter to remove cell debris. The cells were then washed and centrifuged at 413× *g* (Eppendorf 5418R, Hamburg, Germany) at 10 °C for 10 min and suspended in 1 mL of incomplete medium [91]. The number of lymphocytes was calculated using a Burker Cell Counter after Trypan Blue staining.

#### 4.3.3. Lymphocyte Phenotyping

Lymphocytes were stained with fluorescein isothiocyanate (FITC) Mouse Anti-Pig CD4a (cat. no. 559585; BD Biosecinesces, Clone 74-12-4) and phycoerythrin (PE )Mouse Anti-Pig CD8a (cat. no. 559584; BD Biosciences, Clone 76-2-11). Cells were incubated at 4 °C for 15 min, washed with FACS buffer (PBS: 0.1 M phosphate buffered saline, pH = 7.2 supplemented with 5% foetal bovine serum), and centrifuged at 10 °C for 10 min and 413× *g* (Eppendorf 5418R). The supernatant was removed and the cells were fixed in 2% paraformaldehyde and analysed using a MoFloTM XDP flow cytometer (Beckman Coulter Inc., Miami, Fullerton, CA, USA) equipped with a 488 nm air-cooled argon laser. Forward scatter, side scatter, and green and red fluorescence channels were used to collect specific multi-parameter data from the cells. A total of 50,000 events were collected from each sample. The data were analysed with the Summit 5.2 (Beckman-Coulter Inc., Miami, FL, USA) software package. The gating tree was set as follows: forward-scattered light (FSC)/side-scattered light (SSC) (represent distribution cells on size and intracellular composition) lymphocytes were gated in the range 100–150 kDa, followed by CD4+ and CD8+ [92].

### 4.4. Measurement of Serum Concentration of Hp, IL-6, and TNF-α

The serum concentrations of Hp, IL-6, and TNF-α were quantified by commercial the enzyme-linked immunosorbent assay (ELISA) kits:

- Pig Haptoglobin ELISA (HAPT-9, Life Diagnostic Inc., West Chester, PA, USA); sensitivity range from 18.75 to 300 ng/mL
- Quantizing^®^ ELISA Porcine IL-6 (P6000B, R&D Systems, Minneapolis, MN, USA); sensitivity range from 0.68 to 4.30 pg/mL
- Quantizing^®^ ELISA Porcine TNF-α (PTA00, R&D Systems, Minneapolis, MN, USA); sensitivity range from 2.8 to 5.0 pg/mL

For all analyses, serum samples were tested in duplicate, according to the manufacturer’s recommendations. These assays employed the quantitative sandwich enzyme immunoassay technique. A monoclonal antibody specific for a particular protein was pre-coated onto a microplate. The standards, control, and samples were pipetted into the wells and, if it was present, it was bound by the immobilized antibody. After washing away any unbound substances, an enzyme-linked monoclonal antibody specific for peptides was added to the wells. Following a wash step, a substrate solution was added to the wells. After stopping the enzyme reaction, the intensity of the colour was measured. The sample values were then read off the standard curve.

### 4.5. Brain Sample Preparation and Determination of DA Concentrations

Quantitative determination of DA in SN and in PFC was carried out using a commercially available Dopamine ELISA kit (RE59161 IBL International; Hamburg, Germany) according to previously described protocols [93,94]. Briefly, brain samples were weighed and homogenized with an extract solution containing acetonitrile (0.5 mL/100 mg brain tissue), 0.1 M HCl (0.4 mL/100 mg brain tissue), 27 mM ethylenedinitrilotetraacetic acid (EDTA) water solution (0.1 mL/100 mg tissue), and SIGMAFAST Protease Inhibitor Cocktail Tablet EDTA free (cat. no. S8830; Sigma-Aldrich, St. Louis, MO, USA). The sample was then centrifuged at 4500× *g* (Eppendorf 5804) and the supernatant was filtered on syringe filters without pre-filtering (Millex-HV Filter, 0.45 µm, PVDF, Millipore, Burlington, MA, USA). Samples were concentrated on a miVac centrifugal vacuum concentrator, model DNA-23050-800, with SpeedTrap (Genevac Limited, Ipswich, UK) for 2 h, and then lyophilized using an ALPHA 1-4 LSC freeze dryer (MARTIN CHRIST Gefriertrocknungsanlagen GmbH Germany, Osterode am Harz, Germany). Lyophilized samples were stored at −80 °C until analysis. A dopamine ELISA kit was then used according to the manufacturer’s recommendations. First, DA extraction from all samples (unknown, standards, and controls) was done on 24 wells plates with extraction, shaking, and releasing steps (all reagents provided in the kit) followed by ELISA (protocol provided in manual). An Infinite 200 spectrophotometer (Tecan Group, Männedorf, Switzerland) with Magellan software was used to read samples absorbance at a wavelength of 450 nm and calculate dopamine concentration in unknown samples. The results were presented as the average per group ± SD.

### 4.6. Purification and Determination of Neuropeptide Levels from scLN

The levels of GAL, NPY, SP, and VIP were determined in the scLN in the three-step procedure described below.

#### 4.6.1. Sample Preparation and High-Temperature Extraction

Brain peptide extracts from tissues were prepared according to the Conlon procedure [95]. Briefly, frozen tissue was cut into small pieces and 10 mL of hot 1 M acetic acid was added per gram tissue and boiled for 5 min. The samples were then homogenized using Ultra Turax IKA T-25 (Jankel & Kunkel IKA, Germany) at RT for 5 min and centrifuged at 4 °C for 40 min at 4500× *g* (Eppendorf 5804). The supernatant was subject to solid-phase extraction (SPE) step.

#### 4.6.2. Solid-Phase Extraction (SPE) and Concentration

The supernatants were filtered through syringe filters with a graduated glass fibre pre-filter (Millex-HPF HV Filter, 0.45 μm, PVDF, Millipore). Trifluoroacetic acid (TFA) was added to filtrates to obtain a final concentration of 0.1% (*v*/*v*). A Sep-Pak Plus Light Cartridge (130 mg of C18 sorbent per cartridge, Waters, Milford, MA, USA) was used according to the producer’s protocol using a Baker Vacuum Manifold SPE-12G unit (J.T.Baker, Germany). Samples were concentrated on a miVac centrifugal vacuum concentrator, model DNA-23050-800, with SpeedTrap (Genevac Limited, UK) for 2h, and then lyophilized using an ALPHA 1-4 LSC freeze dryer (MARTIN CHRIST Gefriertrocknungsanlagen GmbH Germany). Lyophilized samples were stored at −80 °C until analysis.

All chemicals used were of commercial origin with high performance liquid chromatography (HPLC) grade purity: glacial acetic acid (cat. no. 951503, J.T. Baker), trifluoroacetic acid–TFA (cat. no. 9470, J.T. Baker) and acetonitrile–LC-MS reagent (cat. no. 9821.1000, J.T. Baker, Germany).

#### 4.6.3. Enzyme-Linked Immunosorbent Assay for Quantitative Determination of GAL, NPY, SP, and VIP in scLN

For quantitative determination of GAL, NPY, SP, and VIP in scLN, the following commercial ELISA tests were used: Galanin (GAL) EIA kit, 0–10 ng/mL (S-1210; Peninsula Laboratories International, Inc., San Carlos, CA, USA); Neuropeptide Y (NPY) EIA kit, 0–100 ng/mL (EK-049-03CE; Phoenix Pharmaceuticals, Inc., Burlingame, CA, USA); Substance P EIA kit, 0–5 ng/mL (S-1180; Peninsula Laboratories International, Inc., San Carlos, CA, USA); and Vasoactive Intestinal Peptide (VIP) EIA kit, 0–25 ng/mL (EK-064-16CE; Phoenix Pharmaceuticals, Inc., Burlingame, CA, USA). The manufacturers provided reagents for each assay and protocols (temperatures of incubation, sample volume, reagent volumes) and we followed them. All tests were based on the standard sandwich ELISA method. Briefly, samples together with primary antibodies were put on plates and incubated at room temperature. The plate was then washed and conjugated antibodies were added, and the plates were incubated. After washing, a one-step substrate reagent was added to each well and the plates were incubated again at room temperature. The reaction was terminated with a stop reagent. The absorbance was read at λ = 450 nm on a microplate spectrophotometer Infinite 200 (Tecan) for each sample.

A four-parameter ELISA curve was prepared for each determined neuropeptide (an Excel sheet was provided by Peninsula Laboratories service). Each sample was assayed in duplicate and the peptide concentration was read from the curve. Each peptide concentration was presented as the mean from group ± SD per g of tissue.

### 4.7. Statistical Analysis

The results of body weight and rectal temperature were compared between the control and LPS group using Student’s *t*-test at a significance level of *p* < 0.05. The other results were analysed statistically using a one-way analysis of variance (ANOVA) and the significance of differences between groups was determined using Tukey’s test at a significance level of *p* < 0.05. The data were expressed as mean values ± SD and the calculations were performed with SigmaPlot 12 (Systat Software Inc., Cracow, Poland)

## 5. Conclusions

In conclusion, the results of this study indicate that subclinical LPS from *S.* Enteritidis can affect cells and signal molecules involved in the neuroimmune interaction. We demonstrated increasing levels of DA in the brain and neuropeptides in cLN with a decrease in plasma CD4 and CD8 T-lymphocyte levels after subclinical *S.* Enteritidis LPS administration. Moreover, our data indicate that LPS increases serum Hp levels seven days after *S.* Enteritidis LPS administration. It is possible that even such low doses of LPS from *S.* Enteritidis that do not result in any clinical symptoms of the disease may require eradication, which may be very important, especially in connection with the asymptomatic carrier state of *Salmonella* spp. Understanding how asymptomatic LPS from *S.* Enteritidis impact neuroimmune transmitters and neuroimmune T-lymphocytes responses may be useful for the prevention of pathological processes. Additionally, the results of our study and our considerations in this paper may be helpful in assessing the long-term consequences of low doses of LPS from *S.* Enteritidis. This is especially important in the era of using LPS in developing drugs ranging from vaccines and cancer therapy to immunostimulants, which are becoming increasingly common in use.

## Abbreviations

AD	Alzheimer’s disease
BBB	blood brain barrier
BCSFB	blood-cerebrospinal fluid barrier
cLN	cervical lymph nodes
CSF	cerebrospinal fluid
DA	dopamine
dcLN	deep cervical lymph nodes
GAL	galanin
Hp	haptoglobin
IL-6	interleukin-6
LN	lymph nodes
LPS	lipopolysaccharide
NPY	neuropeptide Y
PD	Parkinson’s disease
PFC	prefrontal cortex
scLN	superficial cervical lymph nodes
SN	substantia nigra
SP	substance P
TNF-α	tumour necrosis factor α
VIP	vasoactive intestinal peptide

## References

[1] De Martel C., Ferlay J., Franceschi S., Vignat J., Bray F., Forman D., Plummer M. (2012). Global burden of cancers attributable to infections in 2008: A review and synthetic analysis. Lancet Oncol..

[2] Mesri E.A., Feitelson M.A., Munger K. (2014). Human viral oncogenesis: A cancer hallmarks analysis. Cell Host Microbe.

[3] Vedham V., Divi R.L., Starks V.L., Verma M. (2014). Multiple Infections and Cancer: Implications in Epidemiology. Technol. Cancer Res. Treat..

[4] Mikołajczyk A. (2017). Invited Brief Commentary on the Article “Breast Cancer Association with Cytomegalo Virus—A Tertiary Center Case-Control Study” Is Cytomegalo Virus a Breast Cancer Etiologic Risk Factor?. J. Investig. Surg..

[5] Zloza A. (2018). Viruses, bacteria, and parasites—Oh my! A resurgence of interest in microbial-based therapy for cancer. J. Immunother. Cancer.

[6] Ramachandran G. (2014). Gram-positive and gram-negative bacterial toxins in sepsis. Virulence.

[7] Liu M., Bing G. (2011). Lipopolysaccharide animal models for Parkinson’s disease. Parkinsons. Dis..

[8] Hoban D.B., Connaughton E., Connaughton C., Hogan G., Thornton C., Mulcahy P., Moloney T.C., Dowd E. (2013). Further characterisation of the LPS model of Parkinson’s disease: A comparison of intra-nigral and intra-striatal lipopolysaccharide administration on motor function, microgliosis and nigrostriatal neurodegeneration in the rat. Brain Behav. Immun..

[9] Sharma N., Nehru B. (2015). Characterization of the lipopolysaccharide induced model of Parkinson’s disease: Role of oxidative stress and neuroinflammation. Neurochem. Int..

[10] Huang B., Liu J., Ju C., Yang D., Chen G., Xu S., Zeng Y., Yan X., Wang W., Liu D. (2017). Licochalcone A prevents the loss of dopaminergic neurons by inhibiting microglial activation in lipopolysaccharide (LPS)-induced Parkinson’s disease models. Int. J. Mol. Sci..

[11] Nguyen M.D. (2004). Exacerbation of Motor Neuron Disease by Chronic Stimulation of Innate Immunity in a Mouse Model of Amyotrophic Lateral Sclerosis. J. Neurosci..

[12] Choi D., Liu M., Hunter R.L., Cass W.A., Pandya J.D., Patrick G., Shin E., Kim H., Gash D.M., Bing G. (2009). Striatal Neuroinflammation Promotes Parkinsonism in Rats. PLoS ONE.

[13] Ma M., Ren Q., Yang C., Zhang J.C., Yao W., Dong C., Ohgi Y., Futamura T., Hashimoto K. (2017). Antidepressant effects of combination of brexpiprazole and fluoxetine on depression-like behavior and dendritic changes in mice after inflammation. Psychopharmacology.

[14] Zhang X.Y., Cao J.B., Zhang L.M., Li Y.F., Mi W.D. (2015). Deferoxamine attenuates lipopolysaccharide-induced neuroinflammation and memory impairment in mice. J. Neuroinflamm..

[15] Hawkesworth S., Moore S.E., Fulford A.J.C., Barclay G.R., Darboe A.A., Mark H., Nyan O.A., Prentice A.M. (2013). Evidence for metabolic endotoxemia in obese and diabetic Gambian women. Nutr. Diabetes.

[16] Zhan X., Stamova B., Jin L.W., DeCarli C., Phinney B., Sharp F.R. (2016). Gram-negative bacterial molecules associate with Alzheimer disease pathology. Neurology.

[17] Pretorius E., Page M.J., Mbotwe S., Kell D.B. (2018). Lipopolysaccharide-binding protein (LBP) can reverse the amyloid state of fibrin seen or induced in Parkinson’s disease. PLoS ONE.

[18] Zhao Y., Jaber V., Lukiw W.J. (2017). Secretory Products of the Human GI Tract Microbiome and Their Potential Impact on Alzheimer’s Disease (AD): Detection of Lipopolysaccharide (LPS) in AD Hippocampus. Front. Cell. Infect. Microbiol..

[19] Banks W.A., Gray A.M., Erickson M.A., Salameh T.S., Damodarasamy M., Sheibani N., Meabon J.S., Wing E.E., Morofuji Y., Cook D.G. (2015). Lipopolysaccharide-induced blood-brain barrier disruption: Roles of cyclooxygenase, oxidative stress, neuroinflammation, and elements of the neurovascular unit. J. Neuroinflamm..

[20] Banks W.A., Robinson S.M. (2010). Minimal penetration of lipopolysaccharide across the murine blood-brain barrier. Brain Behav. Immun..

[21] Marques F., Sousa J.C., Coppola G., Falcao A.M., Rodrigues A.J., Geschwind D.H., Sousa N., Correia-Neves M., Palha J.A. (2009). Kinetic profile of the transcriptome changes induced in the choroid plexus by peripheral inflammation. J. Cereb. Blood Flow Metab..

[22] Marques F., Sousa J.C., Coppola G., Geschwind D.H., Sousa N., Palha J.A., Correia-Neves M. (2009). The choroid plexus response to peripheral inflammatory stimulus. BMC Neurosci..

[23] Niehaus I., Lange J.H. (2003). Endotoxin: Is it an environmental factor in the cause of Parkinson’s disease?. Occup. Environ. Med..

[24] Niehaus I. (2010). In vivo Radiodetoxification of Salmonella minnesota Lipopolysaccharides with radio-labeled Leucine Enkephalin cures sensory polyneuropathy: A Case report. Niger. Health J..

[25] Yao Z., Mates J.M., Cheplowitz A.M., Hammer L.P., Phillips G.S., Wewers M.D., Rajaram M.V.S., John M., Anderson C.L., Ganesan L.P. (2016). Blood-Borne Lipopolysaccharide Is Rapidly Eliminated by Liver Sinusoidal Endothelial Cells via High-Density Lipoprotein. J. Immunol..

[26] Franco R.F., de Jonge E., Dekkers P.E., Timmerman J.J., Spek C.A., van Deventer S.J., van Deursen P., van Kerkhoff L., van Gemen B., ten Cate H. (2000). The in vivo kinetics of tissue factor messenger RNA expression during human endotoxemia: Relationship with activation of coagulation. Blood.

[27] Maxwell J.R., Ruby C., Kerkvliet N.I., Vella A.T. (2002). Contrasting the Roles of Costimulation and the Natural Adjuvant Lipopolysaccharide during the Induction of T Cell Immunity. J. Immunol..

[28] Haudek S.B., Natmessnig B.E., Fürst W., Bahrami S., Schlag G., Redl H. (2003). Lipopolysaccharide dose response in baboons. Shock.

[29] Arredouani M., Matthijs P., Van Hoeyveld E., Kasran A., Baumann H., Ceuppens J.L., Stevens E. (2003). Haptoglobin directly affects T cells and suppresses T helper cell type 2 cytokine release. Immunology.

[30] Arredouani M.S., Kasran A., Vanoirbeek J.A., Berger F.G., Baumann H., Ceuppens J.L. (2005). Haptoglobin dampens endotoxin-induced inflammatory effects both in vitro and in vivo. Immunology.

[31] Mikołajczyk A., Gonkowski S., Złotkowska D. (2017). Modulation of the main porcine enteric neuropeptides by a single low-dose of lipopolysaccharide (LPS) *Salmonella* Enteritidis. Gut Pathog..

[32] Lambrecht B.N. (2001). Immunologists getting nervous: Neuropeptides, dendritic cells and T cell activation. Respir. Res..

[33] Farzi A., Reichmann F., Holzer P. (2015). The homeostatic role of neuropeptide Y in immune function and its impact on mood and behaviour. Acta Physiol..

[34] Wasowicz K., Winnicka A., Kaleczyc J., Zalecki M., Podlasz P., Pidsudko Z. (2018). Neuropeptides and lymphocyte populations in the porcine ileum and ileocecal lymph nodes during postnatal life. PLoS ONE.

[35] Ganea D., Hooper K.M., Kong W. (2015). The neuropeptide VIP: Direct effects on immune cells and involvement in inflammatory and autoimmune diseases. Acta Physiol..

[36] Madva E.N., Granstein R.D. (2013). Nerve-derived Transmitters Including Peptides Influence Cutaneous Immunology. Brain Behav. Immun..

[37] Huang Y., Qiu A.W., Peng Y.P., Liu Y., Huang H.W., Qiu Y.H. (2010). Roles of dopamine receptor subtypes in mediating modulation of T lymphocyte function. Neuroendocrinol. Lett..

[38] Sarkar C., Basu B., Chakroborty D., Dasgupta P.S., Basu S. (2010). The immunoregulatory role of dopamine: An update and. Brain Behav. Immun..

[39] Pacheco R., Contreras F., Zouali M. (2014). The dopaminergic system in autoimmune diseases. Front. Immunol..

[40] Slifstein M., Van De Giessen E., Van Snellenberg J., Thompson J.L., Narendran R., Gil R., Hackett E., Girgis R., Ojeil N., Moore H. (2015). Deficits in prefrontal cortical and extrastriatal dopamine release in schizophrenia a positron emission tomographic functional magnetic resonance imaging study. JAMA Psychiatry.

[41] Ashok A.H., Marques T.R., Jauhar S., Nour M.M., Goodwin G.M., Young A.H., Howes O.D. (2017). The dopamine hypothesis of bipolar affective disorder: The state of the art and implications for treatment. Mol. Psychiatry.

[42] Aspelund A., Antila S., Proulx S.T., Karlsen T.V., Karaman S., Detmar M., Wiig H., Alitalo K. (2015). A dural lymphatic vascular system that drains brain interstitial fluid and macromolecules. J. Exp. Med..

[43] Louveau A., Harris T.H., Kipnis J. (2015). Revisiting the concept of CNS immune privilege Antoine. Trends Immunol..

[44] Liu H., Ni Z., Chen Y., Wang D., Qi Y., Zhang Q., Wang S. (2012). Olfactory route for cerebrospinal fluid drainage into the cervical lymphatic system in a rabbit experimental model. Neural Regen. Res..

[45] Chen L., Elias G., Yostos M.P., Stimec B., Fasel J., Murphy K. (2015). Pathways of cerebrospinal fluid outflow: A deeper understanding of resorption. Neuroradiology.

[46] Laman J.D., Weller R.O. (2013). Drainage of cells and soluble antigen from the CNS to regional lymph nodes. J. Neuroimmune Pharmacol..

[47] Swindle M.M., Makin A., Herron A.J., Clubb F.J., Frazier K.S. (2012). Swine as models in biomedical research and toxicology testing. Vet. Pathol..

[48] Bassols A., Costa C., Eckersall P.D., Osada J., Sabrià J., Tibau J. (2014). The pig as an animal model for human pathologies: A proteomics perspective. Proteom. Clin. Appl..

[49] Pulendran B., Kumar P., Cutler C.W., Mohamadzadeh M., Van Dyke T., Banchereau J. (2001). Lipopolysaccharides from Distinct Pathogens Induce Different Classes of Immune Responses In Vivo. J. Immunol..

[50] Nedrebø T., Reed R.K. (2002). Different serotypes of endotoxin (lipopolysaccharide) cause different increases in albumin extravasation in rats. Shock.

[51] Bryant C.E., Spring D.R., Gangloff M., Gay N.J. (2010). The molecular basis of the host response to lipopolysaccharide. Nat. Rev. Microbiol..

[52] Fedele G., Nasso M., Spensieri F., Palazzo R., Frasca L., Watanabe M., Ausiello C.M. (2008). Lipopolysaccharides from *Bordetella pertussis* and *Bordetella parapertussis* Differently Modulate Human Dendritic Cell Functions Resulting in Divergent Prevalence of Th17-Polarized Responses. J. Immunol..

[53] Mikołajczyk A., Kozłowska A., Gonkowski S. (2018). Distribution and Neurochemistry of the Porcine Ileocaecal Valve Projecting Sensory Neurons in the Dorsal Root Ganglia and the Influence of Lipopolysaccharide from Different Serotypes of *Salmonella* spp. on the Chemical Coding of DRG Neurons in the Cell Cultures. Int. J. Mol. Sci..

[54] Maciel B.M., Rezende R.P., Sriranganathan N.K., Mares M. (2017). Salmonella enterica: Latency. Current Topics in Salmonella and Salmonellosis.

[55] Qin L., Wu X., Block M.L., Liu Y., Breese G.R., Knapp D.J., Crews F.T., Hill C., Carolina N., Park T. (2007). Systemic LPS Causes Chronic Neuroinflammation and Progressive Neurodegeneration. Glia.

[56] Calvano S.E., Coyle S.M. (2012). Experimental Human Endotoxemia: A Model of the Systemic Inflammatory Response Syndrome?. Surg. Infect..

[57] Webel D.M., Finck B.N., Baker D.H., Johnson R.W. (1997). Time course of increased plasma cytokines, cortisol, and urea nitrogen in pigs following intraperitoneal injection of lipopolysaccharide. J. Anim. Sci..

[58] Llamas Moya S., Boyle L., Lynch P.B., Arkins S. (2006). Pro-inflammatory cytokine and acute phase protein responses to low-dose lipopolysaccharide (LPS) challenge in pigs. Anim. Sci..

[59] Maes M. (2011). Depression is an inflammatory disease, but cell-mediated immune activation is the key component of depression. Prog. Neuro-Psychopharmacol. Biol. Psychiatry.

[60] Liu R.H., Pan J.Q., Tang X.E., Li B., Liu S.F., Ma W.L. (2017). The role of immune abnormality in depression and cardiovascular disease. J. Geriatr. Cardiol..

[61] Robertson M.J., Schacterle R.S., Mackin G.A., Wilson S.N., Bloomingdale K.L., Ritz J., Komaroff A.L. (2005). Lymphocyte subset differences in patients with chronic fatigue syndrome, multiple sclerosis and major depression. Clin. Exp. Immunol..

[62] Juffermans N.P., Paxton W.A., Dekkers P.E., Verbon A., de Jonge E., Speelman P., van Deventer S.J., van der Poll T. (2000). Up-regulation of HIV coreceptors CXCR4 and CCR5 on CD4(+) T cells during human endotoxemia and after stimulation with (myco)bacterial antigens: The role of cytokines. Blood.

[63] Palmer C.D., Tomassilli J., Sirignano M., Tejeda M.R., Arnold B., Che D., Lauffenburger D.A., Jost S., Allen T., Mayer K.H. (2014). Enhanced Immune Activation Linked to Endotoxemia in HIV-1 Seronegative Men who have Sex with Men. AIDS.

[64] Guzmán D.C., Herrera M.O., Brizuela N.O., Mejía G.B., Jiménez F.T., García E.H., Olguín H.J. (2016). Assessment of the effects of oseltamivir and indomethacin on dopamine, 5-HIAA, and some oxidative stress markers in stomach and brain of Salmonella typhimurium-infected rats. Neuroendocrinol. Lett. Vol..

[65] Gołembiowska K., Wardas J., Noworyta-Sokołowska K., Kamińska K., Górska A. (2013). Effects of adenosine receptor antagonists on the in vivo lps-induced inflammation model of parkinson’s disease. Neurotox. Res..

[66] Noworyta-Sokolowska K., Gorska A., Golembiowska K. (2013). LPS-induced oxidative stress and inflammatory reaction in the rat striatum. Pharmacol. Rep..

[67] Booij L., Welfeld K., Leyton M., Dagher A., Boileau I., Sibon I., Baker G.B., Diksic M., Soucy J.P., Pruessner J.C. (2016). Dopamine cross-sensitization between psychostimulant drugs and stress in healthy male volunteers. Transl. Psychiatry.

[68] Weinstein J.J., Weinstein J.J., Chohan M.O., Slifstein M., Kegeles L.S., Moore H., Abi-dargham A. (2017). Pathway-Specific Dopamine Abnormalities in Schizophrenia Review Pathway-Speci fi c Dopamine Abnormalities in Schizophrenia. Biol. Psychiatry.

[69] Pacheco R., Contreras F., Prado C., Gowder S. (2012). Cells, molecules and mechanisms involved the neuro-immune interaction. Cell Interaction.

[70] Delgado M., Ganea D. (2000). VIP and PACAP inhibit activation induced apoptosis in T lymphocytes. Ann. N. Y. Acad. Sci..

[71] Bedoui S., Kawamura N., Straub R.H., Pabst R., Yamamura T., Von Hörsten S. (2003). Relevance of neuropeptide Y for the neuroimmune crosstalk. J. Neuroimmunol..

[72] Hauser G.J., Myers A.K., Dayao E.K., Zukowska-Grojec Z. (1993). Neuropeptide Y infusion improves hemodynamics and survival in rat endotoxic shock. Am. J. Physiol..

[73] Bedoui S., von Hörsten S., Gebhardt T. (2007). A role for neuropeptide Y (NPY) in phagocytosis: Implications for innate and adaptive immunity. Peptides.

[74] Trejter M., Brelinska R., Warchol J.B., Butowska W., Neri G., Rebuffat P., Gottardo L., Malendowicz L.K. (2002). Effects of galanin on proliferation and apoptosis of immature rat thymocytes. Int. J. Mol. Med..

[75] Mignini F., Streccioni V., Amenta F. (2003). Autonomic innervation of immune organs and neuroimmune modulation. Auton. Autacoid Pharmacol..

[76] Johnston M., Zakharov A., Papaiconomou C., Salmasi G., Armstrong D. (2004). Evidence of connections between cerebrospinal fluid and nasal lymphatic vessels in humans, non-human primates and other mammalian species. Cerebrospinal Fluid Res..

[77] Radjavi A., Smirnov I., Derecki N., Kipnis J. (2014). Dynamics of the Meningeal CD4+ T-cell repertoire are defined by the cervical lymph nodes and facilitate cognitive task performance in mice. Mol. Psychiatry.

[78] Brochard V., Combadière B., Prigent A., Laouar Y., Perrin A., Beray-Berthat V., Bonduelle O., Alvarez-Fischer D., Callebert J., Launay J.M. (2009). Infiltration of CD4+ lymphocytes into the brain contributes to neurodegeneration in a mouse model of Parkinson disease. J. Clin. Investig..

[79] Schetters S.T.T., Gomez-Nicola D., Garcia-Vallejo J.J., Van Kooyk Y. (2018). Neuroinflammation: Microglia and T cells get ready to tango. Front. Immunol..

[80] McKenna F., McLaughlin P.J., Lewis B.J., Sibbring G.C., Cummerson J.A., Bowen-Jones D., Moots R.J. (2002). Dopamine receptor expression on human T- and B-lymphocytes, monocytes, neutrophils, eosinophils and NK cells: A flow cytometric study. J. Neuroimmunol..

[81] Lucin K., Wyss-Coray T. (2009). Immune activation in brain aging and neurodegeneration: Too much or too little?. Neuron.

[82] Greifenberg V., Ribechini E., Rößner S., Lutz M.B. (2009). Myeloid-derived suppressor cell activation by combined LPS and IFN-γ treatment impairs DC development. Eur. J. Immunol..

[83] Arreola R., Alvarez-Herrera S., Pérez-Sánchez G., Becerril-Villanueva E., Cruz-Fuentes C., Flores-Gutierrez E.O., Garcés-Alvarez M.E., De La Cruz-Aguilera D.L., Medina-Rivero E., Hurtado-Alvarado G. (2016). Immunomodulatory Effects Mediated by Dopamine. J. Immunol. Res..

[84] Kozina E., Sadasivan S., Jiao Y., Dou Y., Ma Z., Tan H., Kodali K., Shaw T., Peng J., Smeyne R.J. (2018). Mutant LRRK2 mediates peripheral and central immune responses leading to neurodegeneration in vivo. Brain.

[85] Mikołajczyk A. (2016). Safe and effective anaesthesiological protocols in domestic pig. Ann. Warsaw Univ. Life Sci. SGGW Anim. Sci..

[86] Fu H.Q., Yang T., Xiao W., Fan L., Wu Y., Terrando N., Wang T.L. (2014). Prolonged neuroinflammation after lipopolysaccharide exposure in aged rats. PLoS ONE.

[87] Lopes P.C. (2016). LPS and neuroinflammation: A matter of timing. Inflammopharmacology.

[88] Saar L.I. (1962). Lymph Nodes of the Head, Neck and Shoulder Region of Swine. Iowa State Univ. Vet. Digit. Respir..

[89] Jelsing J., Hay-Schmidt A., Dyrby T., Hemmingsen R., Uylings H.B.M., Pakkenberg B. (2006). The prefrontal cortex in the Göttingen minipig brain defined by neural projection criteria and cytoarchitecture. Brain Res. Bull..

[90] Waters W.R., Sacco R.E., Dorn A.D., Hontecillas R., Zuckermann F.A., Wannemuehler M.J. (1999). Systemic and mucosal immune responses of pigs to parenteral immunization with a pepsin-digested Serpulina hyodysenteriae bacterin. Vet. Immunol. Immunopathol..

[91] Mierzejewska D., Mitrowska P., Rudnicka B., Kubicka E., Kostyra H. (2008). Effect of non-enzymatic glycosylation of pea albumins on their immunoreactive properties. Food Chem..

[92] Jun S.M., Ochoa-Repáraz J., Zlotkowska D., Hoyt T., Pascual D.W. (2012). Bystander-mediated stimulation of proteolipid protein-specific regulatory T (Treg) cells confers protection against experimental autoimmune encephalomyelitis (EAE) via TGF-β. J. Neuroimmunol..

[93] Li Q., Wong J.H., Lu G., Antonio G.E., Yeung D.K., Ng T.B., Forster L.E., Yew D.T. (2009). Gene expression of synaptosomal-associated protein 25 (SNAP-25) in the prefrontal cortex of the spontaneously hypertensive rat (SHR). Biochim. Biophys. Acta.

[94] Najmanová V., Rambousek L., Syslová K., Bubeníková V., Šlamberová R., Valeš K., Kačer P. (2011). LC-ESI-MS-MS method for monitoring dopamine, serotonin and their metabolites in brain tissue. Chromatographia.

[95] Conlon J.M. (2007). Purification of naturally occurring peptides by reversed-phase HPLC. Nat. Protoc..

